# Analytical and biological assessment of *Magnolia champaca* L. stem bark: Integrating ATR-FTIR, GC–MS, thrombolytic activity, brine shrimp lethality and molecular docking

**DOI:** 10.1016/j.jgeb.2025.100505

**Published:** 2025-05-13

**Authors:** Md. Mahadi Hasan, As-Sazzad Mahmud Nishan, Most. Humayra Binta Rashid, Bijoy Chandra Ghos, Jaytirmoy Barmon

**Affiliations:** a Department of Pharmacy, Dhaka International University, Bangladesh; b Department of Pharmacy, University of Asia Pacific, Bangladesh; c BCSIR Rajshahi Laboratories, Bangladesh Council of Scientific and Industrial Research (BCSIR), Rajshahi 6206, Bangladesh

**Keywords:** ATR-FTIR, GC–MS, Thrombolytic activity, Brine shrimp lethality bioassay, Molecular docking, *Magnolia champaca* L.

## Abstract

Cancer and cardiovascular disease are two of the most significant health challenges worldwide. These diseases are responsible for morbidity and mortality, leading to a substantial burden on healthcare systems and economic resources. Numerous innovative therapies have been proposed for combating cancer and cardiovascular disease like thrombosis; however, many of these treatments involve synthetic drugs that may have undesirable side effects. Consequently, the current research aimed to explore the medicinal properties of phytochemicals derived from *Magnolia champaca* L. This investigation utilized both *in vitro* and *in silico* approaches. The identification of phytocompounds were employed by using ATR-FTIR and GC–MS. Thrombolytic activity was assayed using human venous blood and cytotoxicity was assessed through lethality test using nauplii of *Artemia salina* and finally the molecular docking analysis was performed using a docking software named AutoDock Vina to clearly understand about the interactions of GC–MS identified secondary metabolites with target proteins. The ATR-FTIR spectrum of *Magnolia champaca* specifies the presence of some alkaloids, polysaccharides, flavonoids as well as glycosidic phytocompounds and GC–MS data identified 23 compounds from crude methanolic extract and among them 2,4-Di-*tert*-butylphenol; 3-Ethyl-3-hydroxyandrostan-17-one; Hexadecanoic acid, methyl ester; Di-n-octyl phthalate were prominent. The *in vitro* thrombolytic assay demonstrated that Trans-Syringin and CHF resulted in significant blood clot lysis, achieving 59.39 ± 1.79 % and 45.66 ± 3.63 % respectively comparing 75.75 ± 2.96 % with standard streptokinase. The brine shrimp bioassay indicated moderate to strong toxicological effect. Specifically, Trans-Syringin and the ethyl acetate fraction (EAF) demonstrated considerable cytotoxicity, showing the net values of LC_50_ were 22.39 µg/ml and 61.78 µg/ml, respectively contrary to 11.45 µg/ml of vincristine sulfate (standard). Molecular docking analysis were conducted to explore the synergistic effects of the compounds identified through GC–MS analysis on tissue plasminogen activator and human topoisomerase-II. The compounds 3-Ethyl-3-hydroxyandrostan-17-one; Cadina-1(10),4-diene and 1,1,7-Trimethyl-4-methylenedecahydro-1H-cyclopropa[e]azulen-7-ol-, (1aR-(1a.alpha.,4a.alpha.,7.beta.,7a.beta.,7b.alpha.))- demonstrated high binding affinities of −7.7, −7.3, and −7.1 kcal/mol, respectively, for tissue plasminogen activator where streptokinase (standard) had −6.5 kcal/mol. For human topoisomerase-II, 3-Ethyl-3-hydroxyandrostan-17-one; Caryophyllene-(I1) and Cadina-1(10),4-diene exhibited binding affinities of −8.0, −7.9 as well as −7.8 kcal/mol correspondingly, while standard vincristine had −8.1 kcal/mol. These findings suggest that *Magnolia champaca* contains active phytochemicals with significant potentials for diverse pharmacological applications.

## Highlights

• ATR-FTIR and GC–MS analysis was performed with crude methanolic extract (CME) of *Magnolia champaca* L. stem bark.
• Among the crude extract, the chloroform fraction and the previously isolated compound Trans-Syringin showed significant thrombolytic activity.
• The previously isolated compound Trans-Syringin and EAF had potential cytotoxicity that reveals their activity against neoplastic tumor growth.
• Molecular docking of GC–MS identified compounds with thrombus and tumor forming two proteins was revealed.

## Graphical abstract

**Figure:**
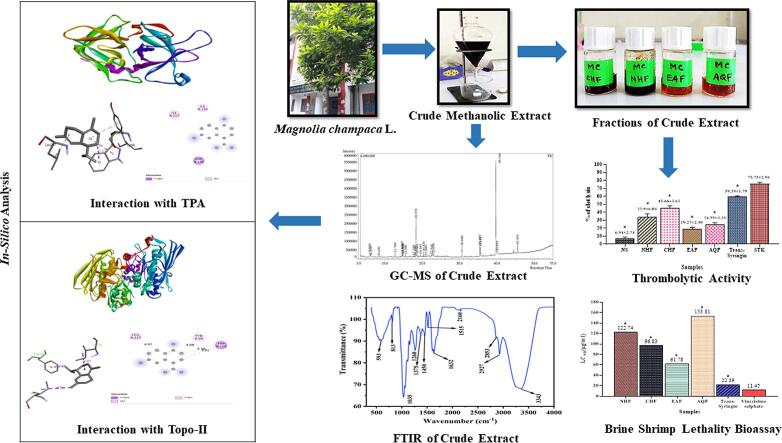
Graphical abstract of the current research

## 1 Introduction

Plants have potential health benefits and used as source of medications from the long decade of human civilization. Natural medicines are the alternative source of synthetic modern medicine and are popular for less side effect, cheaper and effectiveness. In many developing countries, a significant portion of the total inhabitants relies on conventional and herbal medicaments for their everyday healthcare needs involving the utilization of crude medicinal plant extracts containing a wide range of compounds with potential biological activities.^1^ Bark, leaf, flower, fruit and root extracts are rich in nutrition and nutraceuticals and are the main source of medicine. Epidemiologic data reveal that the consumption of these plant extracts responsible for the reduction of the risk of infection, inflammation, cancer, cardiovascular disorders as well as many different chronic diseases.^2^, ^3^

Plants are rich in phytochemicals of different classes such as alkaloids, glycosides, phenolics, steroids, flavonoids and saponins having different biological activities.^4^ Among all the phytochemicals, flavonoid and phenolic compounds have studied most because they are able to neutralize free radicals, scavenge ROS and decompose peroxides and these are occurred by the donation of a hydrogen atom or an electron as those phytochemicals have hydroxyl groups. For that reason, they are called natural antioxidant and are effective in the repair and prevention of oxidative damage of DNA and cell.^5^, ^6^

Many free radicals are liberated from cell during metabolism process and these reactive oxygen species are responsible for destroying cell components and develops many chronic diseases such as diabetes, cancer, coronary artery disease (CAD).^7^ Ethnopharmacological data reveals that malignant cells contain a large number of ROS which accelerates carcinogenesis by promoting DNA and protein damage within cells.^8^ Plant extract acts as natural medicine and prevents malignancy by protecting cellular damage and repairing DNA.^9^ However, much of the information about medicinal herbs available to consumers lacks reliable scientific evidence. Therefore, research is conducted to assess the toxicity of these plants. Researchers need to understand both the toxic effects and potential anticancer properties of plant extracts to fully appreciate their beneficial uses.^1^ Thrombosis is an acute disorder occurs when blood clots block arteries or veins and cumber blood flow in body circulatory pathway. It can be life-threatening causing ischemic heart disease, stroke, heart attack, myocardial infarction and other CVS diseases.^10^ The mortality rate due to blood clotting diseases is stratospheric and heparin, warfarin, streptokinase are currently using in treatment.^11^ Natural medicine can be a good choice besides modern synthetic medicine for their affordability and safety profile to treat thrombosis.^12^ *Magnolia champaca* L., belongs to Magnoliaceae family and locally known as Champak/Champaka is found in Bangladesh, China, Malaysia, Nepal, Indonesia.^13^ The plant is traditionally used for fever, colic, leprosy and rheumatism. The leaf, fruit, bark, flower of M*agnolia champaca* L. possess a large number of reported activities against cancer, infection, inflammation, diabetes, ulcer and other ailments.^14^, ^13^, ^15^, ^16^ Phytochemical investigations of *Magnolia champaca* L. plant parts revealed the existence of glycosides, steroids, flavonoids, alkaloids and many other chemical constituents.^14^, ^16^, ^17^, ^18^ But there are a few ATR-FTIR, GC–MS data and cytotoxic study of *Magnolia champaca* L. stem bark extract and no evidence found to indicate thrombolytic activity of the stem bark as well as its bioactive compounds responsible for such activity. So, in this study, our goal was to explore the ATR-FTIR analysis, GC–MS analysis, cytotoxic and thrombolytic acivities of different fractions and identified compounds of stem bark of *Magnolia champaca* L. with molecular modeling insights.

## 2 Materials and methods

**Fig. 1:**
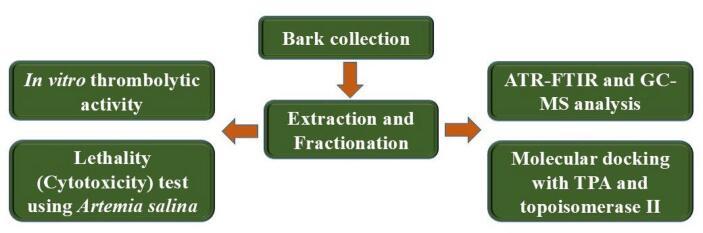
Working procedure of the current research.

In the realm of new drug discovery, both identifying drug binding targets and discovering effective ligands are crucial. This research investigated both *in vitro* and *in silico* methods to assess thrombolytic and cytotoxic activities. Specifically, the study focused on tissue plasminogen activator (TPA) and human topoisomerase-II (TOPO-II) as drug targets because TPA is an epoch-making enzyme that involved in blood clot lysis (fibrinolysis). It triggers plasmin formation from plasminogen and this plasmin is responsible for breaking down of fibrin leading to stroke, deep vein thrombosis, heart attack etc. management.^19^, ^20^ On the other hand, topoisomerase-II is a crucial enzyme which has critical role in replication, transcription and repair of DNA. It involves in DNA strand break temporarily and rejoin the strands which is required for cell division leading to malignancy.^21^, ^22^ The GC–MS identified phytochemicals from CME of *Magnolia champaca* L*.* stem bark was used as potential ligands. Therefore, the goal was to identify possible drug candidates for treating thrombosis and cancer. The detailed working procedure of the current study is demonstrated in Fig. 1.

### 2.1 Chemicals and reagents

Streptokinase, 0.9 % NaCl saline were collected from Incepta Pharmaceuticals Ltd and Vincristine Sulphate from Beacon Pharmaceuticals Ltd., Bangladesh. N-hexane, DMSO, acetoxyethane (ethyl acetate), methyl alcohol (methanol) and chloroform were procured from Merck, Germany. All other required chemicals, reagents and testing agents were pure and analytical grade and procured from the Roche and Sigma–Aldrich.

### 2.2 Preparation of the plant extract

*Magnolia champaca* L. was the experimental plant and the vernal stem bark of matured plant was collected from the botanical garden of University of Rajshahi, Bangladesh. The plant was authenticated (DACB accession number: 48087) by the expert taxonomist of Bangladesh National Herbarium, Dhaka. The experimental plant part (stem bark) was cleaned with normal tap water and kept under sun light to remove moisture for fourteen days. Then the dried stem bark was kept in warmer (electric oven) for forty-eight hours at 42–45 °C. After that the samples were ground by a grinding machine into powder. The powdered stem bark was extracted by methanol to produce crude methanolic extract (CME). Fractionation of CME were performed with different solvents to produce the n-hexane fraction (NHF), chloroform fraction (CHF), ethyl acetate fraction (EAF) and aqueous fraction (AQF) and these fractions were then utilized for the *in vitro* experiments. A pure compound Trans-Syringin was isolated from CHF of *M. champaca* L. by column chromatography and characterized through NMR which was also used in this current investigation.^23^

### 2.3 ATR-FTIR spectroscopic analysis

The functional groups of CME was determined by using ATR-FTIR (Attenuated Total Reflectance-Fourier Transform Infrared Radiation; Model: L1600300, Spectrum Two, PerkinElmer, UK).^24^ This instrument is linked with PerkinElmer Spectrum IR software version 10.6.2 and has a high linearity room temperature DTGS detector. A tiny drop of CME fraction was directly applied to the diamond prism of an ATR attachment. Spectra were then recorded across 4000 cm^−1^ to 400 cm^−1^ wavenumber range and 5 cm^−1^ resolution. At least four duplicates of each specimen were reserved in order to ensure robust observations.

### 2.4 Sample preparation and instrumentation for gas chromatography mass spectrometry analysis

GC–MS was carried out at BCSIR, Rajshahi, Bangladesh. Sample for analysis was developed by addition of 2 mL methanol (GC-grade) to 20 mg of the extract. After that, the whole mixture was properly mixed using a vortex mixer (VM-1000, Taiwan) and finally filtered through a syringe filter. The test sample was then poured into a GC vial for the analysis.

A SHIMADZU GC–MS QP-2020 system with auto-sampler (AOC-20 s) and auto-injector (AOC-20i) was utilized for the sample analysis to find out the phytocompounds. SH Rxi 5MS Sill columns (30mx0.25 mm; 0.25 µm) were used in this study.^25^ Helium (He) was the carrier gas and the pressure flow to the column was 115.8 kPa while 1.70 mL/min was the air flow rate at 48.0 cm/s of linear velocity. The oven was set to begin at 80.0 °C and hold that temperature for two minutes. After that, it was supposed to rise by 4 °C/min to 180.0 °C (which it also held for two minutes) and then to 280.0 °C, which it finally held for one minute. In splitless injection mode, 230.0 °C was set for injector temperature, 240.0 °C set for ion source temperature and the injection volume to 4.0 µL with a 50 splits ratio. The ionization mass spectroscopic analysis was carried out at 70 eV and mass spectra were recorded for 55.00 min with a solvent cut time of 3.50 min, covering the range of 50 *m*/*z* to 500 *m*/*z*. Different compounds were identified from mass spectra data which were confirmed by comparing with libraries such as NIST08s, NIST08 and NIST14.

### 2.5 Assessment of *in vitro* thrombolytic activity

**Fig. 2:**
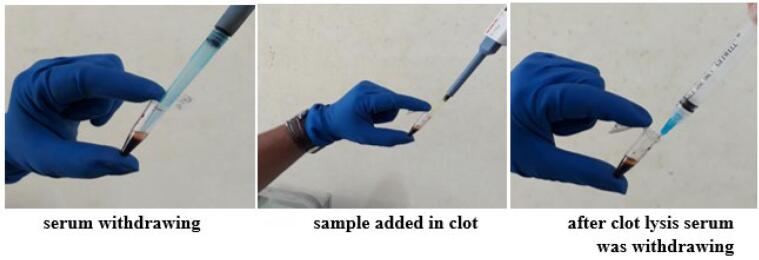
Serum withdrawing and sample added in the clot.

The thrombolytic activities of fractions and compound from *M. champaca* L. stem bark was assessed following a previously described method.^9^, ^26^ Each sample (10 mg) was mixed with 1 ml of saline through vigorous shaking to ensure a homogeneous suspension. Streptokinase was the positive control while normal saline was the negative control. Streptokinase preparation was 15,00,000 IU/vial in 5 mL saline. Venous blood (500 μL) from a healthy human volunteer, free from oral contraceptives or anticoagulants, was transferred into pre-weighed Eppendorf tubes. Then these tubes were kept for incubation for two hours at 37 °C temperature until clot formation occurred. After clotting, the entire blood serum was withdrawn and weight of each blood clot was calculated. To each blood clot containing tube, 100 μL of the respective plant sample (NHF, CHF, EAF, AQF, and Trans-Syringin), standard streptokinase (STK) and normal saline was added separately. After that, the Eppendorf tubes were incubated approximately for 90 min at 37 °C. Finally, the tubes were re-weighed after removing released fluid (Fig. 2) and the clot lysis percentage was calculated using the mentioned formula:

$$Percentage\;\left(\%\right)\;clot\;lysis=\left(\mathrm{Weight}\;of\;\mathrm{clot}\;\mathrm{after}\;\mathrm{lysis}\;/\;Weight\;of\;clot\;before\;lysis\right)\;\times \;100$$

### 2.6 Lethality (cytotoxicity) test using nauplii of *Artemia salina*

The cytotoxicity of fractions and compound derived from *M. champaca* L. stem bark was evaluated using the Brine Shrimp (*Artemia salina*) bioassay.^27^, ^28^ Initially, shrimp eggs were incubated for 48 h in simulated seawater (pH 8.5; temperature 26–30 °C) with continuous aeration to hatch and develop into nauplii (larvae). For each sample (NHF, CHF, EAF, AQF, Trans-Syringin and Vincristine Sulphate), 3 mg was mixed and completely dissolved in 0.6 mL of dimethyl sulfoxide (DMSO) for preparing a stock solution of 5 µg/µL concentration. From these stock solutions, 5, 10, 25, 50, 100, and 200 µL aliquots were added to separate six vials, each containing 10 live nauplii. Seawater was then poured to each vial to bring the final volume to 5 mL, resulting in final sample concentrations of 5 µg/mL, 10 µg/mL, 25 µg/mL, 50 µg/mL, 100 µg/mL, and 200 µg/mL, respectively. After incubation of about 24 h (one day), the test vials were scrutinized under a simple microscope (magnifying glass) to count the total number of surviving nauplii. Mortality rates were calculated for each concentration and LC_50_ values were determined using probit analysis.^29^

### 2.7 Molecular modeling (docking) analysis

#### 2.7.1 Ligand preparation

In this analysis, GC–MS identified 23 compounds, i.e., 1-Butanol, 3-methyl-, formate (PubChem CID: 8052); Benzaldehyde dimethyl acetal (PubChem CID: 62375); Azulene (PubChem CID: 9231); Bicyclo[6.1.0]nona-2,4,6-trien-9-yl methyl ether (PubChem CID: 5370526); (−)-cis-.beta.-Elemene (PubChem CID: 6431151); Cetene (PubChem CID: 12395); Benzenemethanol,.alpha.-methyl-.alpha.-propyl- (PubChem CID: 138214); Caryophyllene (PubChem CID: 5322111); Dodecane, 2,6,11-trimethyl- (PubChem CID: 35768); 3,5-bis(*tert*-butyl)phenol (PubChem CID: 70825); 2,4-Di-*tert*-butylphenol (PubChem CID: 7311); Cadina-1(10),4-diene (PubChem CID: 10223); 2-(3-isopropenyl-4-methyl-4-vinylcyclohexyl)propan-2-ol (PubChem CID: 547972); 1,1,7-Trimethyl-4-methylenedecahydro-1H-cyclopropa[e]azulen-7-ol-, (1aR-(1a.alpha.,4a.alpha.,7.beta.,7a.beta.,7b.alpha.))-, (PubChem CID: 6432640); Caryophyllene-(I1) (PubChem CID: 5369754); Isoelemicin (PubChem CID: 5318557); Guai-1(10)-en-11-ol (PubChem CID: 6432250); Hexadecanoic acid, methyl ester (PubChem CID: 8181); 9,12-Octadecadienoic acid, methyl ester (PubChem CID: 5284421); 9-Octadecenoic acid (Z)-, methyl ester (PubChem CID: 5364509); 3-Ethyl-3-hydroxyandrostan-17-one (PubChem CID: 14681481); Callitrin (PubChem CID: 14038380); Di-n-octyl phthalate (PubChem CID: 8346) and two standards, i.e., Streptokinase (PubChem CID: 9815560) and Vincristine (PubChem CID: 5978) were used. The 3D structure of these ligands was extricated from PubChem database.^30^ After that, the energy of all experimental ligands was optimized by using Avogadro software; version 1.2.0 with the UFF (universal force field). Finally, all ligands were converted into pdbqt format from sdf.

#### 2.7.2 Retrieval and preparation of target protein

Human topoisomerase-II and tissue plasminogen activator were the target protein and their X-ray crystallographic structure were acquired from RCSB protein data bank with PDB ID 1ZXM and 1A5H.^31^ The retrieved proteins were then prepared by cleaning water molecules, ligands and heteroatoms using PyMOL (Academic, version 2.0) and minimized by using Swiss PDB Viewer software version 4.1.^32^, ^33^

#### 2.7.3 Molecular docking of protein-ligand and their visualization

Molecular modeling analysis of the receptors of target protein with the experimental ligands was guided by the ‘Vina Wizard’ program in PyRx software.^34^ At first, the ligand and the macromolecule (protein) were loaded separately into the PyRx tool with proper promulgation of the compound, i.e., ligand or macromolecule. After that, the docked ligand and the optimized target protein were combined by PyMOL. The molecular structure of highest level protein–ligand complexes were viewed with BIOVIA Discovery Studio.^35^ The protein–ligand interactions with different amino acids were investigated and the pictures at the best poses were saved.

### 2.8 Statistical data

The test results were expressed as mean ± SD (standard deviation). Statistical assessments, mathematical calculations and data analysis were conducted by one-way ANOVA following Dunnett’s *t*-test using SPSS (version 20) and GraphPad Prism software (version 8.0.1) where *P < 0.05 = statistically significant. In addition, LC_50_ values were enumerated by Microsoft Excel 2019 using linear regression equations and probit analysis.

## 3 Results

### 3.1 ATR-FTIR analysis

**Fig. 3:**
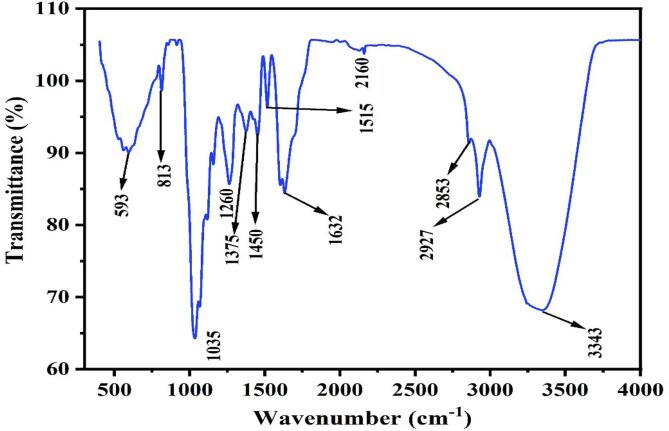
Spectra observed from methanolic extract of *Magnolia champaca* L. stem bark during ATR-FTIR analysis.

Fourier transform infrared transmission is highly helpful in the characterization of plants because it exposes the existence of both organic and inorganic substances in plants. The existence of functional groups in *M. champaca* L. stem bark acts as an indication for the plant's various biological or therapeutic activity. FTIR was used to assess the potential role of *M. champaca* methanolic extract in determining the chemical composition of the organic molecules. The hydroxyl stretching vibration in the range of 3343 cm^−1^ is indicated by a strong broad absorption in the methanolic extract of this plant, as depicted in Fig. 3. This absorption can be attributed to the molecular interaction between the polysaccharide chains. In the same spectral region, it was also observed that the N—H bonds in the amino groups were both symmetrically and asymmetrically stretched which suggests the possibility of alkaloids, which may be brought on by the N—H stretch.^36^ The asymmetric and symmetric CH_2_ stretching vibrations of extract were attributed to the two sharp bands at 2927 cm^−1^ and 2853 cm^−1^, respectively. The signal at 2160 cm^−1^ confirms the presence of C≡C stretching. The signal detected at 1632 cm^−1^ is consistent with the presence of a distorted aromatic ring, amino acids, flavonoids, and C=C group stretching vibrations. It also corresponds to the carbonyl (C=O) stretching vibration. The presence of C=C stretching of the aromatic ring is shown by the peak positions at 1515 and 1450 cm^−1^. The mid-infrared absorption bands in the 1200–800 cm^−1^ range were primarily C—C and C—O stretching vibrations in glycosidic bonds and pyranoid rings, indicating the existence of polysaccharides as the primary component despite their varying structures and compositions. Additionally, C—N stretching signals at 1375 and 1260 cm^−1^ and alkyl halides like C—Br stretching vibrations at 593 cm^−1^.^37^, ^38^ Table 1 lists every probable functional group that is accountable.

**Table 1:** Probable functional groups of *Magnolia champaca* L. stem bark methanolic extract in FTIR-ATR analysis.

Peaks wavenumbers (cm^−1^)	Responsible functional groups
3343	O—H stretching
2927, 2853	C—H stretching
2160	C≡C stretching
1632	C=O stretching
1515, 1450	Aromatic C=C stretching
1375, 1260	C—N stretching
1035, 813	C—O—C stretching
593	C=X stretching

### 3.2 GC–MS identified compounds and their bioactivities

**Fig. 4:**
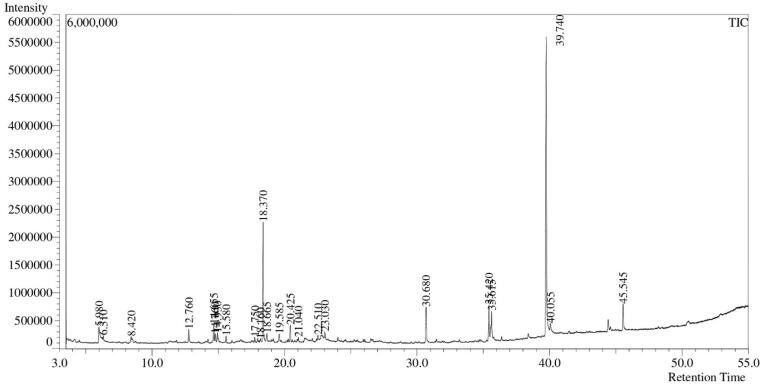
GC–MS data of methanolic extract of *Magnolia champaca* L. stem bark.

There were 23 compounds found through GC–MS of CME of *M. champaca* bark. Fig. 4 shows the chromatogram of identified compounds among them the most significant and prominent compounds were 2,4-Di-*tert*-butylphenol (33.429 %); 3-Ethyl-3-hydroxyandrostan-17-one (18.411 %); Hexadecanoic acid, methyl ester (8.670 %); Di-n-octyl phthalate (6.892 %); 1-Butanol, 3-methyl-, formate (6.243 %) and 9,12-Octadecadienoic acid, methyl ester (4.193 %) presented in Table 2. While the majority of the phytocompounds found in the current study are in line with earlier findings, these compounds relative percentages may differ between reports. Literature says many of the GC–MS identified compounds have pharmacological effectiveness such as antiviral, antibacterial, antifungal, antioxidant, anti-ulcer, anti-inflammatory, antidiabetics, anti-cancer, antiproliferative, immunomodulatory, local anesthetic, analgesics and anti-convulsant activities. On the other hand, some compound had activities against cardiac and neurological diseases.^39^, ^40^, ^41^, ^42^, ^43^, ^44^, ^45^, ^46^, ^47^, ^48^ Literature review about the plant asserted that there was no significant report on GC–MS based *M. champaca* L. stem bark metabolic characterization of its bioactive components. So, this study has explored the very basic baseline data for future studies of the plant stem bark.

**Table 2:** GC–MS identified compounds of *Magnolia champaca* L. stem bark methanolic extract.

ID	Name	R. Time	Area	Conc. (%)	*m*/*z*	SI	S/N
1.	1-Butanol, 3-methyl-, formate	5.980	356,243	6.24337	55.00	98	23.88
2.	Benzaldehyde dimethyl acetal	6.310	103,829	1.81967	121.00	94	62.12
3.	Azulene	8.420	174,584	3.05969	128.00	93	35.46
4.	Bicyclo[6.1.0]nona-2,4,6-trien-9-yl methyl ether	12.760	100,618	1.76339	115.00	98	34.64
5.	(−)-cis-.beta.-Elemene	14.655	96,787	1.69625	93.00	97	25.44
6.	Cetene	14.765	49,853	0.87370	55.00	96	6.98
7.	Benzenemethanol,.alpha.-methyl-.alpha.-propyl-	14.930	63,689	1.11619	147.00	98	16.28
8.	Caryophyllene	15.580	40,245	0.70532	93.00	98	16.41
9.	Dodecane, 2,6,11-trimethyl-	17.750	70,683	1.23876	57.00	96	9.41
10	.3,5-bis(*tert*-butyl)phenol	18.160	45,411	0.79586	191.00	83	25.49
11.	2,4-Di-*tert*-butylphenol	18.370	1,907,445	33.42913	191.00	99	645.30
12.	Cadina-1(10),4-diene	18.665	52,198	0.91480	119.00	95	15.72
13.	2-(3-isopropenyl-4-methyl-4-vinylcyclohexyl)propan-2-ol	19.585	36,597	0.64138	93.00	96	16.58
14.	1,1,7-Trimethyl-4-methylenedecahydro-1H-cyclopropa[e]azulen-7-ol-, (1aR-(1a.alpha.,4a.alpha.,7.beta.,7a.beta.,7b.alpha.))-	20.425	68,143	1.19425	91.00	97	11.58
15.	Caryophyllene-(I1)	21.040	21,794	0.38195	161.00	93	9.00
16.	Isoelemicin	22.510	48,025	0.84167	193.00	95	19.34
17.	Guai-1(10)-en-11-ol	23.030	31,239	0.54748	107.00	95	6.07
18.	Hexadecanoic acid, methyl ester	30.680	494,737	8.67057	74.00	98	123.29
19.	9,12-Octadecadienoic acid, methyl ester	35.420	239,277	4.19347	67.00	98	29.93
20.	9-Octadecenoic acid (Z)-, methyl ester	35.615	215,435	3.77563	55.00	97	19.24
21.	3-Ethyl-3-hydroxyandrostan-17-one	39.740	1,050,574	18.41195	53.00	99	174.61
22.	Callitrin	40.055	45,271	0.79340	68.00	92	10.04
23.	Di-n-octyl phthalate	45.545	393,260	6.89212	149.00	99	99.84

R. Time = Retention Time, *m*/*z* = mass to charge ratio, SI = Similarity Index, S/N = Signal-to-Noise Ratio.

### 3.3 *Ex vivo* thrombolytic activity

**Fig. 5:**
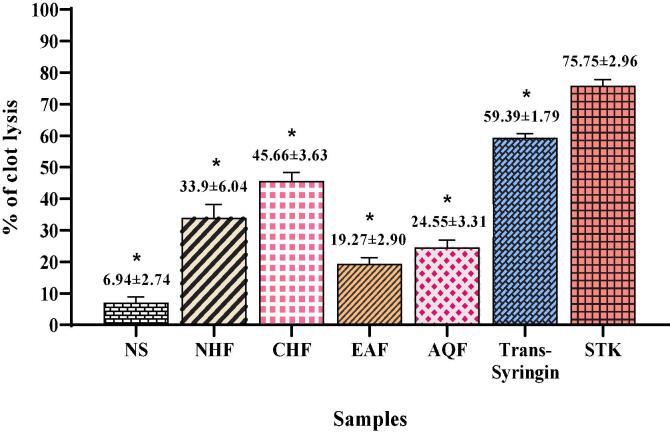
Percentage of clot lysis of Trans-Syringin, NHF, CHF, EAF, AQF, NS and STK.

The weight loss percentage of blood clot after application of test sample solution was considered as the functional indication of *ex vivo (in vitro)* thrombolytic activity. The test result percentage of blood clot lysis of streptokinase, normal saline, one pure compound and different fractions were shown in Fig. 5. Experimental data showed that the compound and different fractions had significant thrombolytic capacity. This assay revealed that Trans-Syringin, NHF, CHF, EAF and AQF fractions showed 59.39 ± 1.79 %, 33.9 ± 6.04 %, 45.66 ± 3.63 %, 19.27 ± 2.90 % and 24.55 ± 3.31 % of blood clot lysis, respectively compared with the standard streptokinase (75.75 ± 2.96 %) and normal saline as negative control (6.94 ± 2.74 %). The order of highest activity was as following:

STK>Trans-Syringin>CHF>NHF>AQF>EAF>NS

### 3.4 Brine shrimp lethality bioassay

**Fig. 6:**
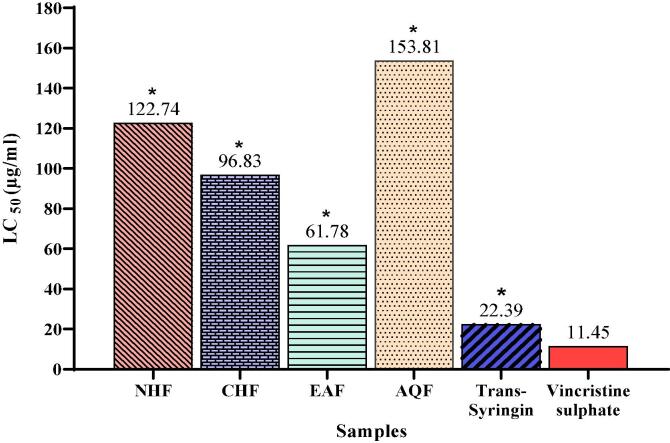
LC_50_ (µg/ml) values of Trans-Syringin and different fractions of CME of *M. champaca* L. stem bark and standard Vincristine Sulphate.

In this experiment, NHF, CHF, EAF, AQF, and Trans-Syringin demonstrated positive results, indicating their biological activity. Particularly, Trans-Syringin and EAF demonstrated notable cytotoxicity, with LC_50_ values of 22.39 µg/mL and 61.78 µg/mL, respectively, compared to vincristine sulphate (standard) with LC_50_ value of 11.45 µg/mL (Fig. 6). The observed cytotoxic effects suggest the presence of potent bioactive compounds within these fractions. Therefore, it is reasonable to infer that Trans-Syringin and EAF may possess potential pesticidal and antitumor properties based on their cytotoxic activities.

### 3.5 Molecular docking result

**Fig. 7:**
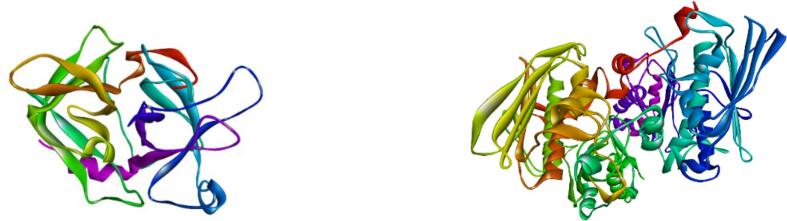
Crystal structure of tissue plasminogen activator (left) and topoisomerase II (right).

**Fig. 8:**
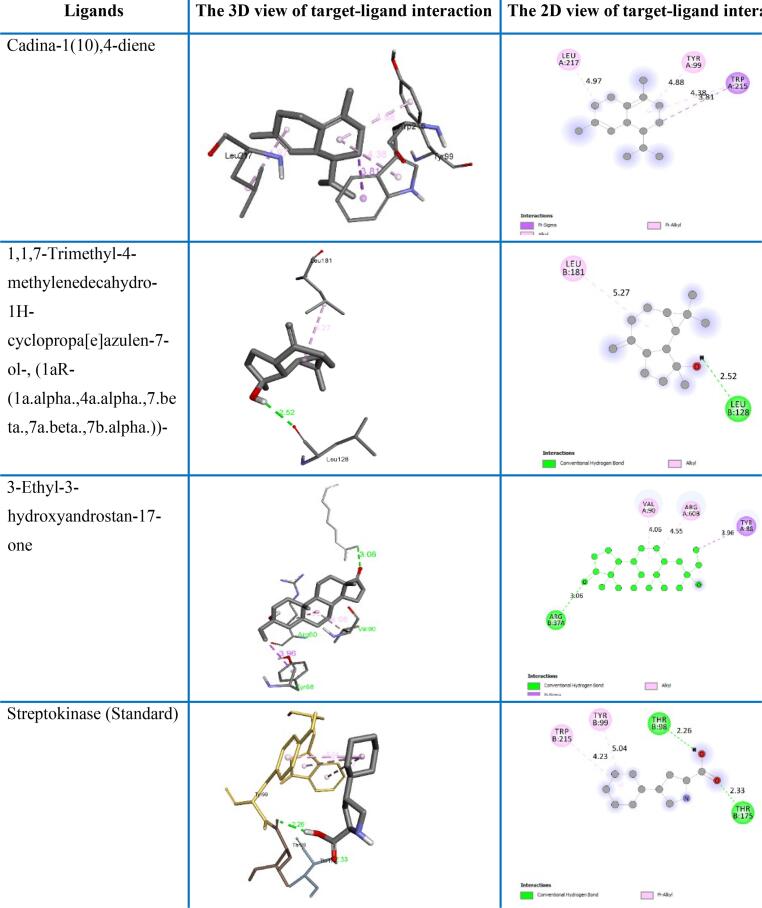
Docking result analysis and ligand–protein binding interaction with tissue plasminogen activator.

**Fig. 9:**
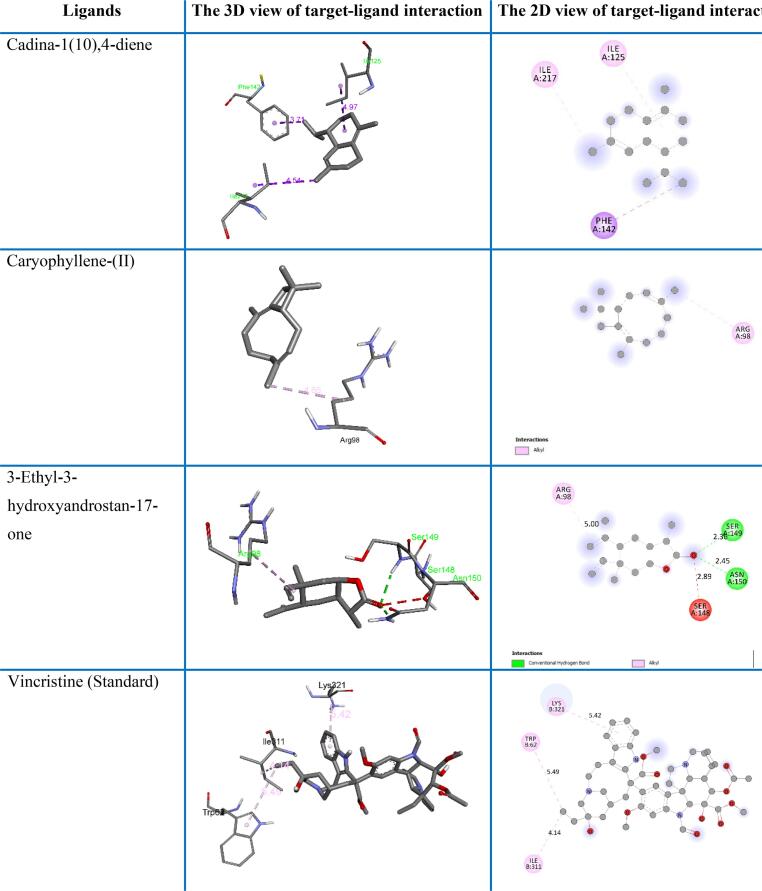
Docking result analysis and ligand–protein binding interaction with topoisomerase II.

The crystal structure of tissue plasminogen activator (TPA) and topoisomerase-II (TOPO-II) is displayed in Fig. 7. Docking score of all 25 compounds (23 compounds from GC–MS data and two standards) interacting with tissue plasminogen activator (TPA) and topoisomerase-II (TOPO- II) are displayed in Table 3. Molecular docking analysis result of three top-ranked ligands (according to binding affinities) and standards are shown in Fig. 8, Fig. 9 and are illustrated in the form of minimum binding energy (kcal/mol) values with interacting amino acids and distance (Table 4, Table 5). Results showed that 3-Ethyl-3-hydroxyandrostan-17-one; Cadina-1(10),4-diene; 1,1,7-Trimethyl-4-methylenedecahydro-1H-cyclopropa[e]azulen-7-ol-, (1aR (1a.alpha.,4a.alpha.,7.beta.,7a.beta.,7b.alpha.))- and standard streptokinase interacted with few amino acids of the tissue plasminogen activator protein with binding affinities of −7.7, −7.3, −7.1 and −6.5 kcal/mol, respectively (Table 4). Here, 3-Ethyl-3-hydroxyandrostan-17-one showed better interaction than others which is comparable to the standard. For the topoisomerase-II target protein, the binding affinities of 3-Ethyl-3-hydroxyandrostan-17-one; Caryophyllene-(I1) and Cadina-1(10),4-diene ligands were −8.0, −7.9 and −7.8 kcal/mol, respectively with some specific amino acids in the protein complex which are comparable with vincristine sulphate having −8.1 kcal/mol binding score (Table 5). Also here, 3-Ethyl-3-hydroxyandrostan-17-one showed better interaction than others which is comparable to the standard. However, 3-Ethyl-3-hydroxyandrostan-17-one and Cadina-1(10),4-diene both interacted better with tissue plasminogen activator and topoisomerase-II. So, it can be claimed that cluster of two compounds could have activities in these diseases or clinical conditions.

**Table 3:** Docking score of all 25 compounds interacting with tissue plasminogen activator (TPA) and topoisomerase-II (TOPO- II).

GC–MS SL.	CID	Name of The Compounds	Docking Score (kcal/mol)	
			TPA	TOPO-II
1	8052	1-Butanol, 3-methyl-, formate	−4.4	−4.5
2	62375	Benzaldehyde dimethyl acetal	−5.3	−5.3
3	9231	Azulene	−6.7	−6.1
4	5370526	Bicyclo[6.1.0]nona-2,4,6-trien-9-yl methyl ether	−5.8	−6
5	6431151	(−)-cis-.beta.-Elemene	−6.3	−7.3
6	12395	Cetene	−4.8	−5.4
7	138214	Benzenemethanol,.alpha.-methyl-.alpha.-propyl-	−5.6	−5.9
8	5322111	Caryophyllene	−6.5	−7.7
9	35768	Dodecane, 2,6,11-trimethyl-	−5.3	−5.5
10	70825	3,5-bis(*tert*-butyl)phenol	−6.3	−6.7
11	7311	2,4-Di-*tert*-butylphenol	−6.3	−6.9
12	10223	Cadina-1(10),4-diene	−7.3	−7.8
13	547972	2-(3-isopropenyl-4-methyl-4-vinylcyclohexyl)propan-2-ol	−6.1	−6.9
14	6432640	1,1,7-Trimethyl-4-methylenedecahydro-1H-cyclopropa[e]azulen-7-ol-, (1aR-(1a.alpha.,4a.alpha.,7.beta.,7a.beta.,7b.alpha.))-	−7.1	−7.5
15	5369754	Caryophyllene-(I1)	−7.1	−7.9
16	5318557	Isoelemicin	−5.4	−6.2
17	6432250	Guai-1(10)-en-11-ol	−6.6	−6.9
18	8181	Hexadecanoic acid, methyl ester	−5.3	−5.4
19	5284421	9,12-Octadecadienoic acid, methyl ester	−5.1	−5.2
20	5364509	9-Octadecenoic acid (Z)-, methyl ester	−5.8	−6.4
21	14681481	3-Ethyl-3-hydroxyandrostan-17-one	−7.7	−8
22	14038380	Callitrin	−6.4	−7.7
23	8346	Di-n-octyl phthalate	−6.3	−6.5
	9815560	Standard (Streptokinase)	−6.5	
	5978	Standard (Vincristin Sulphate)		−8.1

**Table 4:** Non-bonding interaction of compounds with tissue plasminogen activator.

Compound Name	Docking Score (kcal/mol)	Interacting amino acids	Distance	Category	Type
Cadina-1(10),4-diene	−7.3	TRP215	3.81184	Hydrophobic	Pi-Sigma
		LEU217	4.96752		Alkyl
		TYR99	4.87543		Pi-Alkyl
		TRP215	4.38144		
1,1,7-Trimethyl-4-methylenedecahydro-1H-cyclopropa[e]azulen-7-ol-, (1aR-(1a.alpha.,4a.alpha.,7.beta.,7a.beta.,7b.alpha.))-	−7.1	LEU128	2.51892	H-Bond	Conventional H-Bond
		LEU181	5.26637	Hydrophobic	Alkyl
3-Ethyl-3-hydroxyandrostan-17-one	−7.7	ARG37	3.0641	H-Bond	Conventional H-Bond
		TYR88	3.95539	Hydrophobic	Pi-Sigma
		ARG60B	4.5499		Alkyl
		VAL90	4.05782		
Streptokinase (Standard)	−6.5	THR175	2.32575	H-Bond	Conventional H-Bond
		THR98	2.26339		
		TYR99	5.03888	Hydrophobic	Pi-Alkyl
		TRP215	5.15782		
		TRP215	5.14359

**Table 5:** Non-bonding interaction of compounds with topoisomerase II.

Compound Name	Docking Score (kcal/mol)	Interacting amino acids	Distance	Category	Type
Cadina-1(10),4-diene	−7.8	PHE142	3.70612	Hydrophobic	Pi-Sigma
		ILE125	4.97292		Alkyl
		ILE217	4.54159		
Caryophyllene-(I1)	−7.9	ARG98	4.64516	Hydrophobic	Alkyl
3-Ethyl-3-hydroxyandrostan-17-one	−8.0	SER149	2.36013	H-Bond	Conventional H- Bond
		ASN150	2.4535		
		ARG98	5.00241	Hydrophobic	Alkyl
Vincristine (Standard)	−8.1	ILE311	4.14452	Hydrophobic	Alkyl
		TRP62	5.48671		Pi-Alkyl
		LYS321	5.41759		
		ILE311	4.14452		Alkyl

## 4 Discussion

Plants have indeed been crucial sources of therapeutic agents throughout history. Many modern medicines have their origins in traditional plant-based remedies.^49^ Natural products remain a significant focus in drug discovery, even in today's advanced age, due to the incredible variety of biomolecules found in plants. These compounds offer novel pathways for addressing a range of chronic diseases and continue to provide valuable opportunities for developing new therapeutic treatments.^50^ Current treatments for thrombus formation include antiplatelet and anticoagulant drugs such as aspirin, thienopyridine, heparin, warfarin etc. which work by inhibiting blood clot formation. However, these medications have limitations, including reduced effectiveness over extended periods and potential for unforeseen side effects.^51^, ^52^ Consequently, recent research has increasingly focused on alternative drug sources that may present fewer or minimal side effects. Several natural plant species, including *Spatholobus suberectus, Ginkgo biloba, Chaenomeles sinensis, Curcuma longa, Salvia miltiorrhiza, Boswellia serrata, Garcinia nervosa,* Agrimonia Pilosa and *Piper longum* have been studied for their potential thrombolytic properties.^53^, ^54^ Antioxidants, glycosides, alkaloids, and numerous phenolic and flavonoid compounds are documented to show extensive biological activities comprising antimicrobial, anti-nociceptive, anti-inflammatory, anticancer and thrombolytic effects.^55^ In this study, we investigated the characterization of *Magnolia champaca* L. stem bark extract using ATR-FTIR and GC–MS data analysis with *in vitro* and computational approaches to explore possible thrombolytic and cytotoxic activity.

ATR-FTIR was used to elucidate the phytochemical composition of Magnolia shampaca, mainly aiming at the identification of alkaloids, flavonoids, glycosides, polysaccharides, etc.^56^ ATR-FTIR is a valuable analytical tool that provides insight into the molecular structure by measuring vibrational transitions of functional groups, offering a non-destructive and efficient approach to analyze complex plant extracts and provides a complete profile of its phytochemical constituents.^57^ FTIR showed peaks for alkaloids, flavonoids, glycosides, and polysaccharides aligned in good agreement with their known spectral features, validating the use of ATR-FTIR for such studies. This chemical profile is consistent with the pharmacological properties attributed to *Magnolia champaca*, L. suggesting potential therapeutic values.^13^, ^58^

GC–MS test identified a wide range of phytocompounds in the methyl alcohol extract of *Magnolia champaca* L. stem bark. Notably, 2,4-Di-*tert*-butylphenol; 3-Ethyl-3-hydroxyandrostan-17-one; Hexadecanoic acid, methyl ester; Di-n-octyl phthalate; 1-Butanol, 3-methyl-, formate and 9,12-Octadecadienoic acid, methyl ester was identified by its retention time (RT) and mass spectrum which were in agreement with literature data suggesting their activity as major components contributing to its reputed therapeutic properties.^58^ Benzaldehyde dimethyl acetal calming anticancer effects and 2,4-Di-*tert*-butylphenol has antimicrobial and anticancer properties are particularly noteworthy. The presence of caryophyllene suggests potential for anti-inflammatory treatments and neuroprotective benefits.^59^, ^60^ Understanding the chemical profile of *Magnolia champaca* L. helps to clarify its traditional uses and opens up avenues for the development of new therapies. This finding not only confirm the physicochemical profile of *Magnolia champaca* L. but also highlight its potential therapeutic benefits.

The thrombolytic activity assay examined the ability of *Magnolia champaca* L. stem bark fractions and *trans*-syringin to reduce blood clot formation. The results showed considerable thrombolytic activity in specific fractions, with *trans*-syringin showing a more pronounced effect.^61^, ^62^ This observation suggests that some compounds such as *trans*-syringin in *Magnolia champaca* L. may have fibrinolytic properties, which could be valuable for the production of thrombolytic drugs. The observed activity aligns with the traditional uses of *Magnolia champaca* L. in treating cardiovascular disorders.

Bioassay of brine shrimp provided insights into the cytotoxicity of *Magnolia champaca* L. stem bark fractions and *trans*-syringin. The assay revealed different level of toxicity in different fractions and *trans*-syringin showed significant lethality. This bioassay is a preliminary rapid indicator of the anticancer and antiparasitic effects of the compounds.^63^, ^64^ The observed toxicity could be ascribed to biochemicals with detrimental effects on the brine shrimp larvae, suggesting the possibility of further clinical research and pharmacological exploration.

In docking study, we tested the interactions of 23 specific compounds with standard streptokinase, evaluating their binding affinities to the tissue plasminogen activator (TPA) protein. Binding affinity indicates the strength and formation of the docking interactions between compounds and TPA. Higher negative values represent stronger interactions.^65^ This molecular docking study highlights the promising interaction of 3-Ethyl-3-hydroxyandrostan-17-one with ARG37, TYR88, ARG60B and VAL90; interactions of Cadina-1(10),4-diene with TRP215, LEU217 and TYR99; and also interactions of 1,1,7-Trimethyl-4-methylenedecahydro-1H-cyclopropa[e]azulen-7-ol-, (1aR-(1a.alpha.,4a.alpha.,7.beta.,7a.beta.,7b.alpha.))- with LEU128 and LEU181 (Table 4 and Fig. 8). These amino acids are the part of comprehensive morphological structure of TPA and the interactions of compound and amino acids may confer to the stability of enzyme, their binding and specificity in catalytic pathway that directs fibrinolysis.^66^, ^67^ Docking with tissue plasminogen activator, suggesting their potential as novel thrombolytic agents and compounds with higher binding affinities are likely to have a more substantial impact on the TPA’s ability for plasminogen to plasmin conversion, thereby enhancing fibrinolysis and thrombolysis because plasmin is the key ingredients for fibrin and thrombus breakdown. The observed binding affinities can be attributed to specific interactions between these compounds and amino acids in the TPA protein like H-bonding, hydrophobic linkage or van der Waals interactions (Table 4 and Fig. 8).^68^, ^69^

Computational studies to evaluate the cytotoxicity of several compounds with the topoisomerase-II target protein claiming the binding affinities obtained for 3-Ethyl-3-hydroxyandrostan-17-one, Caryophyllene, and Cadina-1(10),4-diene are close to that of vincristine sulfate. Vincristine sulfate is a well-known topoisomerase II inhibitor used in cancer therapy. Its binding affinity serves as a benchmark for assessing the potential effectiveness of the new compounds.^70^, ^71^ Topoisomerase-II is essential for DNA replication for all type of cells. It is a nuclear enzyme that breaks the double-strand in DNA and afterwards the strands are sealed. Improper handling of DNA breaks causing genomic instability leading to cancer with uncontrolled cell proliferation due to overexpression of topoisomerase-II. But, if this enzyme fails to reseal the broken strand properly then the break will be unresolved resulting DNA damage, apoptosis or cell death. Many anticancer drugs work through this mechanism.^72^, ^73^, ^74^

The GC–MS identified compound 3-Ethyl-3-hydroxyandrostan-17-one interacted with SER149, ASN150 and ARG98; Caryophyllene interacted with ARG98; and Cadina-1(10),4-diene interacted with PHE142, ILE125 and ILE217 amino acid residues of the binding pocket of topoisomerase-II with alkyl, Pi-alkyl, Pi-sigma and conventional H-bonding whereas standard vincristine sulphate interacted with ILE311, TRP62 and LYS321 amino acids with alkyl and Pi-alkyl bonding (Table 5 and Fig. 9) which suggests that these compounds may have similar topoisomerase-II inhibitory properties compared to vincristine. These interactions can impede the effectiveness of the macromolecule (enzyme), which is important for its role in DNA replication and cell division.^75^, ^76^, ^77^ Inhibition of TOPO-II enzyme may lead to the DNA damage and cell death in tumor cell. This is one of the key mechanisms of various chemotherapeutic agents.^78^, ^79^ The uncovering of the current study presents a promising basis for further drug development and evaluation of these compounds as potential topoisomerase-II inhibitors. Furthermore, experimental validation and optimization are needed to explore their potential as therapeutic agents.

Some prior investigations revealed that Caryophyllene exhibited anticancer activity,^80^ Cadina-1(10),4-diene showed antibacterial and antifungal activity,^41^ 3-Ethyl-3-hydroxyandrostan-17-one exhibited anticonvulsant activity^48^ and 1,1,7-Trimethyl-4-methylenedecahydro-1H-cyclopropa[e]azulen-7-ol-, (1aR-(1a.alpha.,4a.alpha.,7.beta.,7a.beta.,7b.alpha.)) showed antioxidant, anti-inflammatory, antiproliferative and immunomodulatory activities^42^, ^43^ whereas no reliable data found regarding the molecular docking studies of GC–MS identified 23 compounds with TPA and TOPO-II. Therefore, our current *in silico* study may be considered as first time reported investigation.

The limitations of this study include that only GC–MS analysis was conducted that can identify volatile compounds mainly. Besides, only two proteins tissue plasminogen activator (TPA) and topoisomerase-II are selected for molecular docking. In addition, this research did not focus on the examination of the exact mechanisms through which different fractions of CME demonstrate thrombolytic activity and cytotoxic effects. Further molecular dynamics simulations and structural analyzes are warranted to elucidate the precise sites of interaction of these compounds with TPA and topoisomerase-II. Such studies may lead to the identification of novel thrombolytic and anticancer compounds having improved efficacy and specificity.

## 5 Conclusion

Our current investigation explored that the GC–MS analysis of stem bark of *M. champaca* identified 23 compounds where aromatic, flavonoid, glycosidic and polysaccharide compounds were present and identified through ATR-FTIR. Pure compound Trans-Syringin and chloroform fraction were most prominent to exhibit thrombolytic activity which maybe by triggering the tissue plasminogen activator protein. On the other hand, Trans-Syringin along with ethyl acetate fraction showed notable cytotoxicity leading to antitumor and pesticidal effect which may be due to the inhibition of topoisomerase-II enzyme. The GC–MS identified compounds exhibited promising binding affinities towards the two target proteins in molecular docking studies. The docking analysis validates the thrombolytic and anticancer properties of different fractions of plant stem bark as well as the compounds. Integration of ATR-FTIR, GC–MS, thrombolytic activity, brine shrimp lethality bioassay and molecular docking analysis illustrate a complete understanding for detection of phytochemical and biological properties of *Magnolia champaca* L. stem bark and its key phytocompounds. The findings of the present study highlight *Magnolia champaca* L. as an efficient source of bioactive phytocompounds with considerable therapeutic activity, especially in thrombolytic and anticancer activities. Further studies are needed to investigate and validate these findings, and pave the way for potential therapeutic improvements.

## Ethical approval statement

Approval was not obtained from any organization as there was no animal model used in the study.

## Funding and acknowledgement

All the authors and co-authors did not receive any funding of financial assistantship from any institution to carry out the research.

## CRediT authorship contribution statement

**Md.Mahadi Hasan:** Writing – original draft, Supervision, Project administration, Methodology, Investigation, Formal analysis, Conceptualization. **As-Sazzad Mahmud Nishan:** Writing – review & editing, Software, Investigation. **Most.Humayra Binta Rashid:** Writing – review & editing, Formal analysis. **Bijoy Chandra Ghos:** Software, Resources, Investigation, Formal analysis, Data curation. **Jaytirmoy Barmon:** Validation, Methodology, Investigation, Data curation.

## Declaration of competing interest

The authors declare that they have no known competing financial interests or personal relationships that could have appeared to influence the work reported in this paper.

## Data Availability

The data described in this article are available upon request from the corresponding author.
